# Antarctic Microalga *Chlamydomonas* sp. ICE-L Cryptochrome CiCRY-DASH1 Mediates Efficient DNA Photorepair of UV-Induced Cyclobutane Pyrimidine Dimer and 6-4 Photoproducts

**DOI:** 10.3390/md24010025

**Published:** 2026-01-07

**Authors:** Zhou Zheng, Xinning Pan, Zhiru Liu, Yanan Tan, Zejun Wu, Ning Du

**Affiliations:** 1 Marine Bioresource & Environment Research Center, First Institute of Oceanography, Ministry of Natural Resources, Qingdao 266061, China; 2 Laboratory for Marine Drugs and Bioproducts, Qingdao Marine Science and Technology Center, Qingdao 266237, China

**Keywords:** Antarctic microalga, CiCRY-DASH1 DNA photorepair, UV-induced DNA damage, marine bioactive agents, protein engineering

## Abstract

Cryptochromes (CRYs) are a conserved class of blue light and near-ultraviolet light receptors that regulate diverse processes, including photomorphogenesis in plants. In the extreme Antarctic environment, ice algae endure intense UV radiation, prolonged darkness, and low temperatures, where cryptochromes play a vital role in light sensing and stress response. In this study, we cloned the complete open reading frame (ORF) of the cryptochrome gene CiCRY-DASH1 from the Antarctic microalga *Chlamydomonas* sp. ICE-L. Both in vivo and in vitro DNA photorepair assays showed that CiCRY-DASH1 effectively repairs cyclobutane pyrimidine dimer (CPD) and 6-4 photoproducts (6-4PPs) induced by UV radiation. Furthermore, deletion of the N-terminal and C-terminal loop regions, combined with activity assays, revealed that the C-terminal loop region plays a crucial role in photorepair activity. These findings elucidate the adaptive photorepair mechanisms of Antarctic microalgae and establish CiCRY-DASH1 as a valuable genetic resource. Specifically, the high catalytic efficiency and evolutionary robustness of the engineered variants position it as a promising marine bioactive agent for photoprotective therapeutics and a strategic target for constructing microbial chassis to enable sustainable drug biomanufacturing.

## 1. Introduction

Solar radiation acts as a critical environmental regulator for photosynthetic organisms. While visible light drives photosynthesis and entrains circadian rhythms [1,2,3], the ultraviolet (UV) component imposes significant physiological stress, particularly in aquatic ecosystems [4]. Specifically, UVB radiation (290–320 nm) triggers the formation of cytotoxic DNA lesions, including cyclobutane pyrimidine dimer(CPD) and 6-4 photoproducts(6-4PPs) [5]. The accumulation of these photoproducts compromises genomic integrity and threatens the survival of organisms exposed to high-UV environments, such as those in Antarctica [6]. To counter this light-induced stress, organisms have evolved multiple DNA repair mechanisms [7], including photoreactivation [8].

Cryptochromes, as receptors for blue light and near-UV light [9], are highly conserved across evolution and widely distributed in archaea, bacteria, and eukaryotes [10]. Sharing homologous domains with DNA repair enzymes (photolyases), they thus form the Cryptochrome/Photolyase family (CPF) [11], a class of light-absorbing flavoproteins involved in light sensing, circadian rhythm regulation, and DNA repair across all life forms [10,12,13], which has attracted considerable scientific interest.

DASH-type cryptochromes (CRY-DASH), a specialized member of the CPF, retain the photolyase activity of ancestral proteins while possessing typical cryptochrome signaling functions [14]. Studies have shown that CRY-DASH proteins exhibit functional selectivity under different light conditions: under intense light, they primarily function in DNA repair, whereas under weak light, they act as blue light receptors in signal transduction [15]. This functional diversity makes CRY-DASH an ideal model for studying the evolution of light receptors. However, most research on CRY-DASH has focused on terrestrial organisms and mesophilic algae, with limited attention to extremophiles, particularly Antarctic ice algae, leaving a gap in our understanding of the light adaptation mechanisms of extremophiles.

Antarctica’s unique geographical conditions create an extreme habitat [16]: during the polar day, organisms experience continuous intense solar radiation (light intensity can reach 1700 μmol photons m^−2^s^−1^) [17] and enhanced UVB radiation due to the ozone hole [18]; during the polar night, they endure prolonged darkness and low temperatures [19,20]. Over the long term, Antarctic ice algae have developed specialized physiological adaptations to thrive in this sea ice habitat, characterized by extended photoperiods (18–24 h) and severe physicochemical gradients [21]. Remarkably, these extremophiles exhibit robust psychrophilic and halotolerant traits, capable of maintaining growth even at −7 °C and 115‰ salinity [22]. These distinctive characteristics are well-exemplified by *Chlamydomonas* sp. ICE-L, the specific strain investigated in this study. Despite such harsh conditions, *Chlamydomonas* sp. ICE-L exhibits exceptional adaptability to low temperatures and high salinity, along with a robust light stress response system that protects against intense UV radiation. As key primary producers in the Antarctic sea ice ecosystem, Antarctic ice algae exhibit a direct cellular response to environmental stimuli and are easily cultivated and genetically manipulated, making them an ideal model for extremophilic biology research. The cryptochrome system of ice algae plays a central role in adapting to extreme light cycles and high UV radiation [23], rendering the underlying molecular mechanisms highly valuable for investigation.

In previous work [24], we identified a light-responsive DASH-type cryptochrome CiCRY-DASH1 in *Chlamydomonas* sp. ICE-L. Compared with previously characterized CRY-DASH proteins, CiCRY-DASH1 originates from an extreme Antarctic marine environment, suggesting it may possess superior UV tolerance, biochemical stability, and photorepair robustness. These properties make it particularly attractive for developing marine-derived photoprotective agents. Consequently, the characterization of CiCRY-DASH1 positions it as a promising enzymatic candidate for the development of topical biopharmaceuticals designed to mitigate UV-induced DNA damage and prevent skin carcinogenesis.

To elucidate the molecular mechanisms underlying this adaptation and explore its biotechnological potential, this study integrated AlphaFold structural prediction, prokaryotic expression, and functional assays to characterize CiCRY-DASH1. Given that the extended N-terminal and C-terminal loops are absent in mesophilic homologs, we speculated that these unique structures might function as critical regulatory domains. To verify this, we validated the DNA photorepair activity of the wild-type protein both in vivo and in vitro and systematically investigated the impact of these loop regions on repair function through selective deletions. Beyond revealing the adaptation mechanisms of Antarctic ice algae, this study highlights CiCRY-DASH1 as a promising candidate for marine biotechnological applications. Its robust DNA photorepair capability positions it as a potential enzyme-based therapeutic for mitigating UV-induced DNA damage and preventing skin carcinogenesis. Furthermore, as a key regulator of light-dependent metabolism, CiCRY-DASH1 presents a novel genetic target for optimizing the bioproduction of high-value marine bioactive compounds. Collectively, these findings provide a theoretical and material foundation for advancing both marine drug discovery and sustainable biomanufacturing.

## 2. Results and Discussion

### 2.1. Bioinformatics Analysis of CiCRY-DASH1

Bioinformatic analysis showed that CiCRY-DASH1 encodes an 1836 bp ORF, translates into a 610 amino acid polypeptide. The predicted molecular formula is C_3007_H_4683_N_849_O_872_S_28_, with a theoretical molecular mass of 67.58 kDa and an isoelectric point (pI) of 9.37. The calculated instability index (II) is 45.31, which classifies it as an unstable protein, and the average grand average of hydropathy (GRAVY) score of −0.490 indicates weak hydrophilicity.

**Figure 1:**
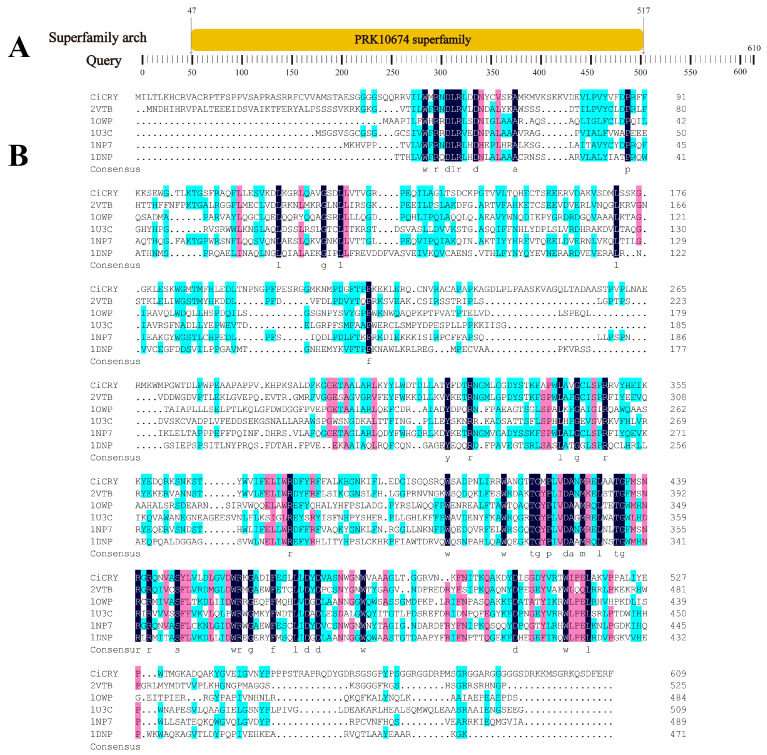
Domain organization and sequence homology of CiCRY-DASH1. (**A**) Protein domain of the CiCRY-DASH1 amino acid sequence; (**B**) amino acid sequence alignment comparison. Note: 1: *Chlamydomonas* sp. ICE-L; 2: *Synechocystis* sp. PCC 6803; 3: *Arabidopsis thaliana* (thale cress); 4: *Synechococcus elongatus* PCC 6301 (*Synechococcus leopoliensis* SAG 1402-1); 5: *Arabidopsis thaliana* (thale cress); 6: *Escherichia coli*.

NCBI BLAST analysis revealed that the amino acid sequence of *CiCRY-DASH1* contains conserved domains of the CPF (Figure 1A). Multiple sequence alignment (Figure 1B) showed the highest sequence similarity with Cryptochrome DASH from *Synechocystis* sp. (SsCRY; PDB: 1NP7) and *Arabidopsis thaliana* (AtCRY; PDB: 2VTB), with sequence identities of 44% and 38%, respectively. Notably, the N-terminal (residues 1–45) and C-terminal (residues 560–610) regions are enriched in glycine (Gly) and serine (Ser) residues. Such enrichment of small polar amino acids is known to hinder the formation of ordered secondary structures, such as α-helices and β-sheets. These glycine/serine-rich loops may confer structural flexibility that could regulate cofactor positioning or DNA-binding dynamics, suggesting potential functional importance.

### 2.2. Construction of CiCRY-DASH1 Recombinant Strains and Protein Expression

**Figure 2:**
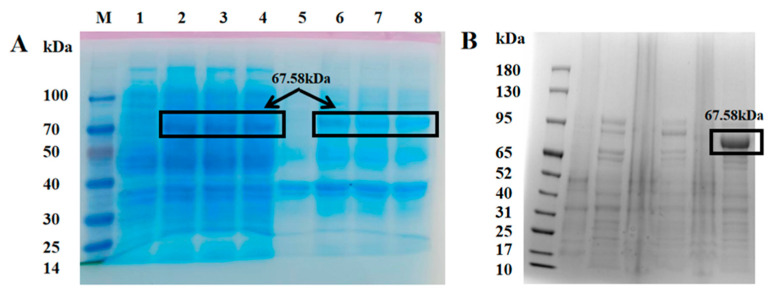
SDS-PAGE Detection of CiCRY-DASH1 Protein Results. (**A**) SDS-PAGE detection of CiCRY-DASH1 protein expressed in *E. coli*; (**B**) SDS-PAGE detection of CiCRY-DASH1 protein purification. Note: M: Marker; 1: No-load bacterial supernatant; 2–4: CiCRY-DASH1 supernatant; 5: No-load bacterial precipitate; 6–8: CiCRY-DASH1 precipitate.

Agarose gel electrophoresis results of the recombinant plasmid pET-28a-CiCRY-DASH1 are shown in Figure S1. After successful construction of the CiCRY-DASH1 expression strain, the protein was heterologously expressed in *E. coli* BL21 (DE3) cells. Following IPTG induction, the recombinant protein was purified by Ni-NTA affinity chromatography with an imidazole gradient. SDS-PAGE analysis revealed a band consistent with the theoretical molecular weight of CiCRY-DASH1 (Figure 2), confirming its expression in *E. coli* BL21 (DE3).

### 2.3. Predicted Tertiary Structure and Comparative Protein Structural Analysis of CiCRY-DASH1

The three-dimensional structure of CiCRY-DASH1 predicted by AlphaFold2 is shown in Figure S2. The five predicted models exhibited highly consistent structural features, with conserved topological conformation of the core region as indicated by Predicted Local Distance Difference Test scores. Predicted Alignment Error heatmap analysis (Figure S3) identified Model 2 as the most structurally reliable, with a global average pLDDT of 84.2, which is higher than that of the other models. Multiple Sequence Alignment analysis (Figure S4) retrieved approximately 13,000 homologous sequences from the database, of which ~38.5% were high-quality templates with over 60% sequence identity to CiCRY-DASH1, providing a robust basis for homology-driven structural modeling.

**Figure 3:**
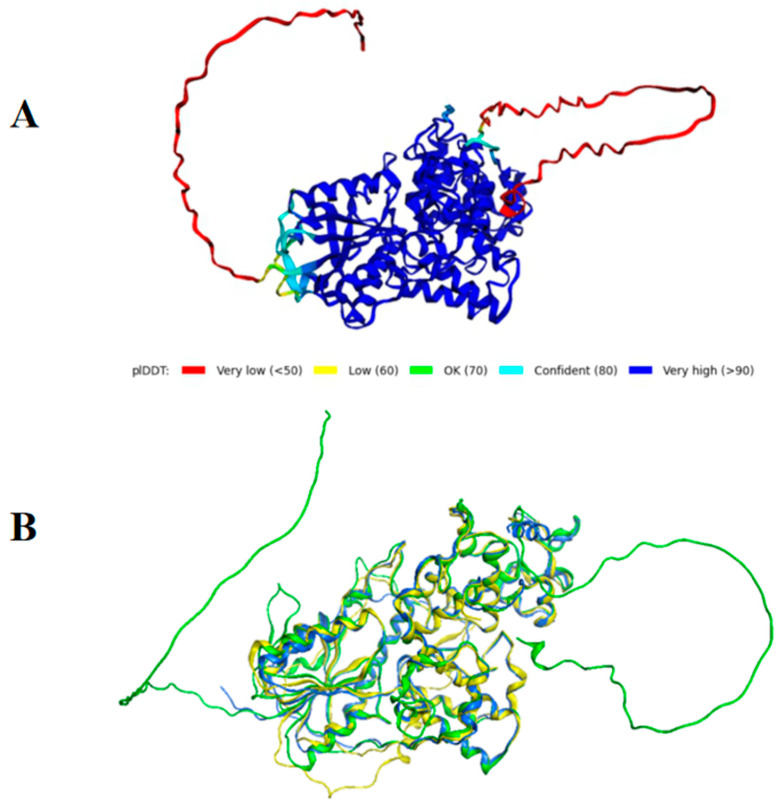
(**A**) The 3D structure of CiCRY-DASH1 predicted by AlphaFold; (**B**) comparison of the AlphaFold predicted structure of CiCRY-DASH1 protein with the structure of other proteins. Note: Green: CiCRY-DASH1; Blue: 1NP7; Yellow: 2VTB.

Local Distance Difference Test results (Figure S5) showed that the five models displayed highly similar confidence profiles in the 50–550 aa core region, with fluctuation patterns strongly correlated with MSA coverage distribution. AlphaFold2 prediction (Figure 3A) indicated that CiCRY-DASH1 adopts a compact overall fold, except for extended disordered loops at the N- and C-termini. Structural comparison with homologous proteins SsCRY (PDB: 1NP7) and AtCRY (PDB: 2VTB) (Figure 3B) revealed conserved core domains. Consistent with this observation, structural alignment further shows that while the catalytic core of CiCRY-DASH1 is highly conserved with the mesophilic photolyase from *Arthrospira platensis* [25], significant divergence exists in the terminal regions. Specifically, our modeling identifies distinct extended loops at both the N- and C-termini, a feature that contrasts with the typically compact terminal structures of mesophilic homologs. Based on these structural characteristics, we hypothesize that these flexible terminal extensions function as dynamic regulatory domains. Similarly to the autoinhibitory mechanisms described in mammalian and plant cryptochromes, these loops likely act as steric ‘gates’ that occlude the catalytic pocket, modulating substrate access and preventing constitutive activation [26].

### 2.4. In Vivo and In Vitro Activity Assays of CiCRY-DASH1

#### 2.4.1. Activity Assay Results of CiCRY-DASH1 in *E. coli*

**Figure 4:**
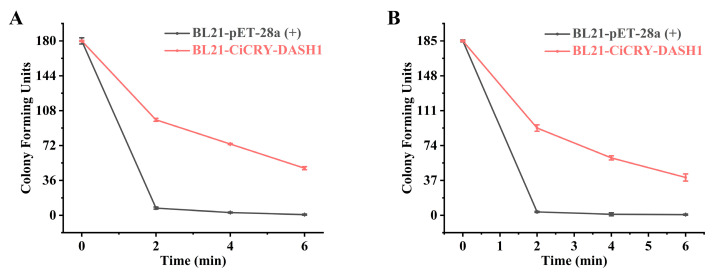
In vivo survival analysis of *E. coli* strains under UV irradiation. (**A**) Bacterial survival quantified by colony-forming unit(CFU) counts under photoreactivation (light repair) condition. (**B**) Bacterial survival CFU counts under dark repair conditions.

To evaluate the in vivo DNA repair efficacy of CiCRY-DASH1, bacterial survival was quantified using colony-forming unit (CFU) counts (Figure 4A,B). The control strain (BL21-pET-28a) exhibited acute sensitivity to UV radiation, with viability collapsing to near-detection limits (<10 CFU) within just 2 min. In distinct contrast, cells expressing CiCRY-DASH1 maintained robust survival. Under photoreactivation conditions, the CiCRY-DASH1 strain retained approximately 99 CFU and 49 CFU after 2 and 6 min of exposure, respectively. While maximal repair efficiency was light-dependent, significant protection was also observed in the absence of light. The dark-incubated cells maintained 93 CFU at 2 min and 40 CFU at 6 min—significantly higher than the control. This data confirms that while CiCRY-DASH1 functions primarily as a light-activated DNA photolyase, it confers a baseline level of UV tolerance independent of illumination.

#### 2.4.2. Assay of CiCRY-DASH1 Repair Activity Towards CPD Under White and Blue Light

**Figure 5:**
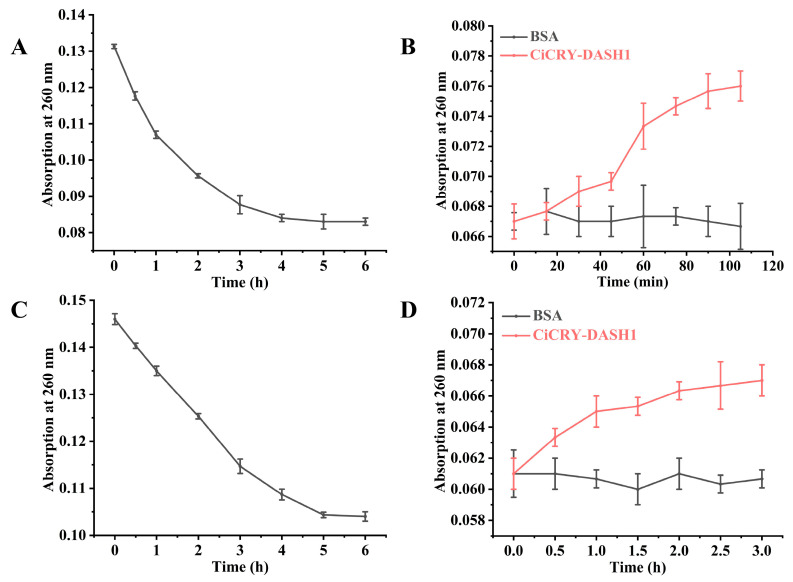
Detection of cyclobutane pyrimidine dimer(CPD) repair activity by CiCRY-DASH1 under white and blue light. (**A**) The change in light absorption value of pyrimidine dimer when irradiated with UVB. (**B**) Change in light absorption value of pyrimidine dimer with cryptochrome addition under white light. (**C**) The change in light absorption value of pyrimidine dimer when irradiated with UVB. (**D**) Change in light absorption value of pyrimidine dimer with cryptochrome addition under blue light.

To prepare the CPD-containing substrates, Oligo(dT)_16_ single-stranded DNA was subjected to UVB irradiation. As shown in Figure 5A,C, the A_260_ value progressively decreased with prolonged UV exposure, indicating the successful formation of CPD. Irradiation was maintained until the absorbance reached a stable minimum, ensuring a standardized level of DNA damage across samples for the subsequent photorepair assays.

The repair kinetics of CiCRY-DASH1 were subsequently characterized by monitoring the A_260_ recovery under both white and blue light. In control groups treated with BSA, the absorbance remained stagnant at the damaged baseline, confirming that the DNA monomerization was strictly enzyme-dependent and ruled out non-enzymatic repair. In contrast, the addition of CiCRY-DASH1 triggered a notable increase in absorbance within the initial 100 min, representing a rapid repair phase. Following this burst, the reaction rate exhibited a characteristic deceleration as it approached a steady-state plateau (Figure 5B,D).

For instance, the CRY-DASH from the green alga *Ostreococcus tauri* exhibits specific binding and repair capabilities toward CPD-damaged DNA [27]. Similarly, the red alga *Cyanidioschyzon merolae* possesses two homologs, CmPHR2 and CmPHR5, capable of monomerizing CPD lesions [28]. While CRY-DASH proteins in *Volvox carteri* and *Chlamydomonas reinhardtii* have been identified, their biochemical repair efficiencies remain to be fully elucidated [29]. Notably, CRY-DASH maintains a high catalytic efficiency comparable to the ssDNA-specific repair observed in *Arthrospira platensis* [25]. As the first characterized CRY-DASH from an Antarctic microalga, the rapid repair kinetics of CiCRY-DASH1 suggest a specialized adaptation to the persistent UV stress of the polar marine environment, distinguishing it from mesophilic counterparts. Such performance underscores its potential as a promising candidate for development in marine-based photoprotective therapeutics and biotechnological applications.

### 2.5. Assay of CiCRY-DASH1 Repair Activity for 6-4PPs

**Figure 6:**
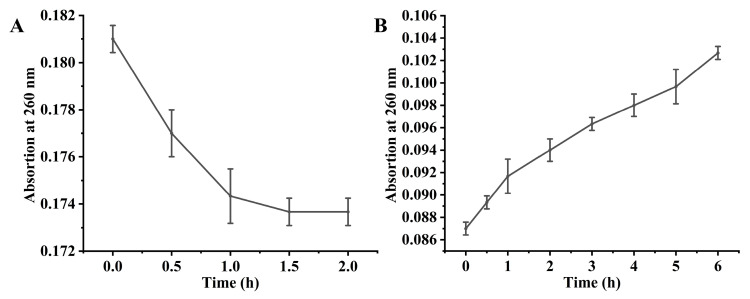
Detection of repair activity of 6-4 photoproducts(6-4PPs) by CiCRY-DASH1 under blue light. (**A**) Change in 6-4PPs absorbance under UVB irradiation. (**B**) Change in 6-4PPs absorbance with cryptochrome addition.

To evaluate the repair activity toward 6-4PPs, a specific DNA substrate (5′-GTATTATG-3′) was synthesized. As shown in Figure 6A, the A_260_ value exhibited a steady decrease under UVB irradiation, confirming the successful induction of 6-4PPs lesions. Subsequently, the photorepair process was initiated by introducing CiCRY-DASH1 under blue light (450–460 nm). The results revealed a continuous and progressive recovery of A_260_ throughout the 6 h incubation period (Figure 6B), indicating that CiCRY-DASH1 effectively monomerizes 6-4PPs lesions.

These findings confirm that CiCRY-DASH1 is a bifunctional marine bioactive substance capable of repairing both CPD and 6-4PPs lesions. Such versatile DNA repair activity underscores its biological significance in maintaining the genomic stability of *Chlamydomonas* sp. ICE-L within the extreme, high-UV environment of its Antarctic habitat.

### 2.6. The Effect of the Loop Region of CiCRY-DASH1 on Photoreactivation Activity

#### 2.6.1. Construction of Truncated Loop Region Proteins

Truncated CRY proteins were constructed, and the plasmid information for the four proteins is shown in Figure S6 and Table S2. Sequencing results of the plasmids are presented in Figures S7–S10. The sequencing data were consistent with the design, confirming that the constructs are suitable for subsequent experiments.

#### 2.6.2. Protein Expression and Purification

**Figure 7:**
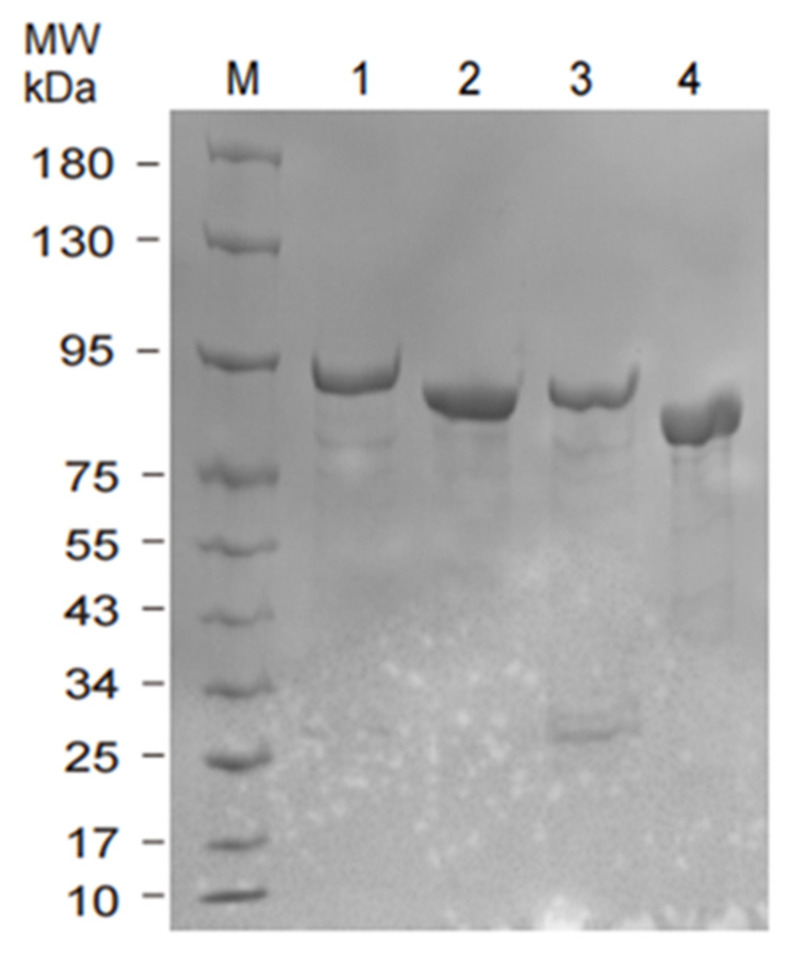
SDS-PAGE detection of four truncated CRY proteins. Note: M: Marker; 1: CRY(1–610); 2: CRY(46–610); 3: CRY(1–560); 4: CRY(46–560).

After transforming the four plasmids into *E. coli*, all four recombinant 6His-GST-CRY proteins (CRY1–610, CRY46–610, CRY1–560, and CRY46–560) were solubly expressed under low-temperature induction at 16 °C. Small-scale purification tests indicated that all fusion proteins efficiently bound to glutathione resin, and PreScission Protease cleavage successfully released protein fragments of the expected sizes (Figures S11–S14). Following Ni-NTA purification, SDS-PAGE analysis (Figure 7) revealed a single major band for each protein, consistent with the theoretical molecular weight, confirming the successful expression and purification of the proteins.

#### 2.6.3. CPD Repair Experiment with CRY Proteins

**Figure 8:**
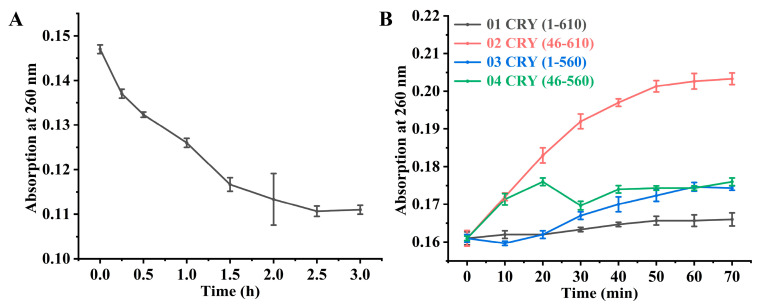
Truncated CRY protein-mediated CPD repair activity. (**A**) Change in pyrimidine dimer absorbance under UVB irradiation; (**B**) Change in pyrimidine dimer absorbance with the addition of different CRY proteins.

UVB irradiation induced CPD formation in Oligo(dT)_16_ single-stranded DNA, with the A_260_ value continuously decreasing (Figure 8A). After three hours of irradiation, approximately 24.49% of DNA molecules were damaged, forming CPD. In vitro photorepair experiments conducted under uniform light conditions (Figure 8B) revealed varying increases in A_260_ after adding the four truncated CRY proteins, with maximum values achieved approximately 70 min after addition.

The repair kinetics varied significantly among the truncated variants, indicating distinct regulatory roles for the terminal extensions. Full-length CRY (1610) exhibited a steady but modest recovery in absorbance. In stark contrast, the N-terminal truncated variant CRY (46–610) triggered a rapid surge in A_260_ within the initial 50 min, ultimately achieving the highest repair efficiency among all tested constructs. Deletion of the C-terminal loop CRY (1–560) or both termini CRY (46–560) also yielded enhanced repair capacities relative to the wild-type, characterized by pronounced initial bursts followed by kinetic stabilization. These profiles suggest that the removal of terminal disordered regions effectively ‘unlocks’ the enzyme’s catalytic potential.

In summary, the CPD photorepair capacities of the truncated variants CRY (46–610), CRY (1–560), and CRY (46–560) were markedly superior to those of the full-length CRY (1–610). These findings demonstrate that the terminal loops of CiCRY-DASH1 function as intrinsic modulators of function. The superior performance of CRY (46–610) suggests that the N-terminal extension may impose steric hindrance, restricting substrate access to the catalytic pocket; thus, its excision likely optimizes protein dynamics and substrate-binding occupancy. As a novel marine bioactive substance, the enhanced efficiency of these engineered variants provides a solid molecular foundation for developing marine-based photoprotective agents to mitigate UV-induced DNA damage

#### 2.6.4. 6-4PPs Repair Experiment with CRY Proteins

**Figure 9:**
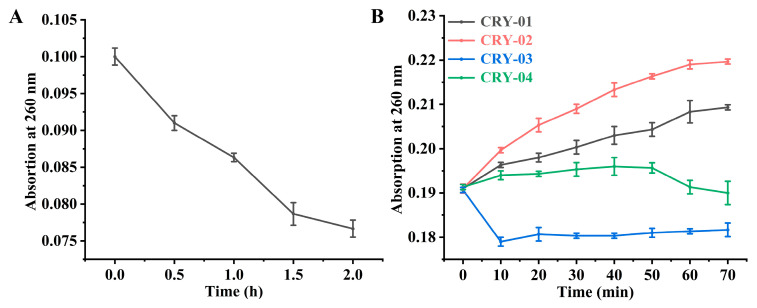
Detection of repair activity of 6-4PPs by CRY protein. (**A**) The change in 6-4PPs light absorption value when irradiating UVB; (**B**) Changes in light absorption values of 6-4 PPS after adding different CRY proteins.

To evaluate the repair efficiency of the truncated variants toward 6-4PPs, a specific DNA substrate (5′-GTATTATG-3′) was prepared via UVB irradiation (Figure 9A). Subsequent photorepair assays under blue light revealed significant functional divergence among the variants (Figure 9B). While the full-length CRY (1–610) exhibited a steady increase in absorbance, the N-terminal truncated variant CRY (46–610) showed a rapid and superior repair trajectory, achieving a 15.01% increase in A_260_ relative to the initial value within 70 min. This substantial enhancement underscores the inhibitory nature of the N-terminal extension, where its removal likely facilitates more efficient substrate access to the catalytic center. In stark contrast, the C-terminal deletion CRY (1–560) resulted in a complete loss of 6-4PPs repair activity, with the A_260_ failing to recover throughout the incubation. The double-truncated variant CRY (46–560) displayed only marginal recovery, with a minimal A_260_ increase of 2.27%. These results indicate that while the N-terminal loop acts as a regulatory constraint, the C-terminal loop is indispensable, specifically for 6-4PPs monomerization. This substrate-specific requirement suggests that the C-terminal region is essential for stabilizing the ‘base flipping’ mechanism. As proposed by Sancar [30], the repair process requires the DNA lesion to be flipped out of the duplex and into the enzyme’s hydrophobic catalytic pocket; for the structurally distorted 6-4PPs, the C-terminal loop likely provides the necessary structural scaffold to facilitate this energetically demanding transition. Collectively, CRY (46–610) emerged as the most robust variant, with its functional enhancement mirroring the autoinhibition mechanisms reported in plant cryptochromes [31]. As a novel marine bioactive substance, the identification of these opposing regulatory roles for the terminal loops provides a strategic molecular blueprint for the rational design of advanced photoprotective enzymes.

### 2.7. Potential Biotechnological Applications in Marine Drug Discovery and Biomanufacturing

The functional characterization and successful engineering of CiCRY-DASH1 variants in this study extend the implications of our findings from ecological adaptation to practical marine biotechnology. Beyond their established roles in DNA photorepair, CPF members are increasingly regarded as promising candidates for photoprotective and dermatological applications. Photolyases, which share close mechanistic homology with CRY-DASH proteins, have already been incorporated into topical formulations and evaluated in clinical and preclinical settings. These studies report beneficial effects in reducing UV-induced DNA lesions, actinic keratoses, and photoaging [32,33], demonstrating the safety and feasibility of using DNA repair enzymes in skin-directed therapies. Moreover, CRY/photolyase homologs from marine and Antarctic microorganisms often display exceptional UV tolerance and biochemical stability [34]. In addition to their physiological roles, marine-derived enzymes are increasingly recognized as high-value bioactive macromolecules with significant pharmaceutical potential. Distinct from conventional small-molecule drugs, enzyme-based therapeutics represent an emerging frontier in marine pharmacology.

Regarding therapeutic applications, the identification of the hyperactive variant CRY46–610 provides empirical evidence that Antarctic-derived enzymes are amenable to optimization through rational design. Given that high-efficiency DNA photolyases are currently deployed in topical medical devices to mitigate UV-induced DNA damage and prevent the progression of actinic keratosis [32,33], the significantly enhanced catalytic efficiency observed in CRY46–610 is particularly relevant. Coupled with the exceptional robustness derived from its evolutionary adaptation to the extreme Antarctic environment, this engineered variant represents a promising candidate for the development of novel marine-derived therapeutic agents.

Furthermore, in the context of biomanufacturing, the structural data obtained herein offer specific genetic targets for metabolic engineering. Cryptochromes serve as central regulators of the circadian clock, which gates the metabolic flux of carbon toward secondary metabolites. Modulating CRY-mediated light signaling is an established strategy to promote cell growth from metabolite accumulation, thereby facilitating the production of high-value bioactive compounds in microalgal cell factories [35]. Consequently, these findings provide strong theoretical support for the sustainable production of marine-derived pharmaceuticals.

## 3. Materials and Methods

### 3.1. Algae Culture

The Antarctic microalga *Chlamydomonas* sp. ICE-L was isolated from sea ice near Zhongshan Station (69°22′ S, 76°22′ E) during the 18th Chinese National Antarctic Research Expedition (2001–2002) [36] and cryopreserved in the Marine Biological Resource Bank of the First Institute of Oceanography, Ministry of Natural Resources (Qingdao, China). For experimental cultivation, the strain was inoculated into sterile seawater-based Provasoli’s medium [37] (600 mL in 1 L Erlenmeyer flasks) and maintained at 5 °C under a 12 h light:12 h dark photoperiod with an irradiance of 40 μmol photons m^−2^ s^−1^. The seawater medium had a salinity of 32‰, and flasks were manually swirled by hand three times daily (morning, noon, and evening) to ensure uniform growth.

### 3.2. Strains, Plasmids, and Culture Medium

*E. coli* BL21 (DE3) and pET-28a(+) were purchased from Tiangen (Beijing, China). The components of Provasoli medium are detailed in Table S1. Luria–Bertani (LB) medium contained 10.0 g of tryptone, 5.0 g of yeast extract, and 10.0 g of NaCl per liter of distilled water. LB medium was supplemented with 1.5% agar. All media were sterilized at 121 °C for 20 min.

### 3.3. Reagents and Materials

Protein and DNA molecular weight markers were purchased from TransGen Biotech (Beijing, China). Kanamycin, Ampicillin and isopropyl-β-D-thiogalactoside (IPTG) were purchased from Solarbio Co., Ltd. (Beijing, China). All chemical reagents were of analytical grade. Primers, DNA Oligo(dT)_16_, and the primer 5′-GTATTATG-3′ were synthesized by Sangon Biotech (Shanghai, China).

### 3.4. RNA Extraction and cDNA Synthesis

Approximately 50 mL of *Chlamydomonas* sp. ICE-L cultures in the logarithmic growth phase were harvested by filtration through 0.22 μm membrane filters. Total RNA was extracted from liquid nitrogen-ground algal powder using TRIzol reagent (TransGen Biotech, Beijing, China) following the manufacturer’s instructions. RNA integrity was verified by 1% agarose gel electrophoresis, and concentration and purity were determined using a NanoVue Plus spectrophotometer (GE Healthcare, Chicago, IL, USA). cDNA was synthesized using TransScript^®^ One-Step gDNA Removal and cDNA Synthesis SuperMix (TransGen Biotech, Beijing, China) and stored at −20 °C for subsequent experiments.

### 3.5. Cloning and Sequencing of CiCRY-DASH1 Complete ORF

Based on the previously established transcriptome database of *Chlamydomonas* sp. ICE-L, the CiCRY-DASH1 cDNA sequence was identified and retrieved. Gene-specific primers (*CiCRY-DASH1*-F: ATGATATTGACCCTGAAGCATTGC and *CiCRY-DASH1*-R: TCCAAAGCGCTCGAAGTCGC) were designed using Primer Premier 5.0 software and synthesized by Sangon Biotech (Shanghai, China).

PCR amplification of the CiCRY-DASH1 cDNA fragment was performed under the following conditions: initial denaturation at 98 °C for 1 min; 35 cycles of denaturation at 98 °C for 10 s, annealing at 58 °C for 5 s, and extension at 72 °C for 10 s. PCR products were separated by 1% agarose gel electrophoresis, and the target band was excised and purified using the EasyPure Quick Gel Extraction Kit (TransGen Biotech, Beijing, China). The purified fragment was cloned into the pEASY^®^-Blunt Zero Cloning Vector (TransGen Biotech) and transformed into *E. coli* Trans1-T1 competent cells (TransGen Biotech).

Positive transformants were selected on LB agar plates supplemented with 100 μg/mL ampicillin. Five randomly chosen colonies were cultured in LB medium containing 100 μg/mL ampicillin, and DNA sequencing was performed by Sangon Biotech (Shanghai, China).

### 3.6. Bioinformatic Characterization of CiCRY-DASH1

Comprehensive bioinformatic analyses were performed to characterize CiCRY-DASH1. Sequence homology was assessed using NCBI BLAST (Web server; https://blast.ncbi.nlm.nih.gov/Blast.cgi, accessed on 20 November 2025), and the coding sequence was translated to amino acids using the ExPASy Translate tool (Web server; https://web.expasy.org/translate/, accessed on 20 November 2025). Physicochemical properties of the deduced protein were predicted with ProtParam (Web server; https://web.expasy.org/protparam/, accessed on 20 November 2025), and potential phosphorylation sites were identified with NetPhos 3.1 (Web server; https://services.healthtech.dtu.dk/services/NetPhos-3.1/, accessed on 28 October 2025) [38]. Domain architecture analysis was conducted via CD-search (Web server; http://www.ncbi.nlm.nih.gov/Structure/cdd/wrpsb.cgi, accessed on 10 November 2025), and multiple sequence alignments were carried out using DNAMAN software (Version 9.0; Lynnon Biosoft, San Ramon, CA, USA)

### 3.7. Construction of CiCRY-DASH1 Recombinant Engineering Strains and Protein Expression

The CiCRY-DASH1 coding sequence was amplified from *Chlamydomonas* sp. ICE-L cDNA using high-fidelity PCR with gene-specific primers (*CiCRY-DASH1*-eF: GGAATTCCATATGCATATGATCCTGACCCTGAAGC and *CiCRY-DASH1*-eR: CCGCTCGAGCTCGAGTTAGCCGAATCGCTC). The amplicon was cloned into the pET-28a(+) expression vector via *NdeI* and *XhoI* restriction sites, generating the recombinant plasmid pET28a-CiCRY-DASH1 with an N-terminal 6×His tag. Positive clones were verified by sequencing (Sangon Biotech). The recombinant plasmid was transformed into *E. coli* BL21(DE3) competent cells. BL21-pET28a-CiCRY-DASH1 strains and the empty vector BL21-pET28a were cultured under identical induction conditions.

Single colonies were inoculated into LB medium supplemented with 50 μg/mL kanamycin and cultured at 37 °C with shaking (160 rpm) until the OD_600_ reached 0.6–0.8 (approximately six h). IPTG was added to a final concentration of 0.5 mM, and protein expression was induced at 16 °C with shaking (160 rpm) for 8 h. Cells were harvested by centrifugation at 6000× *g* for 10 min at 4 °C, resuspended in PBS, and lysed using an ultrasonic cell disrupter. The soluble fraction containing the recombinant protein was collected and purified using a Ni-NTA His Tag Kit (GE Healthcare, Uppsala, Sweden) with an imidazole gradient (10–500 mM). Eluted protein fractions were analyzed by sodium dodecyl sulfate-polyacrylamide gel electrophoresis (SDS-PAGE).

### 3.8. Tertiary Structure Prediction and Protein Structure Comparison of CiCRY-DASH1

The tertiary structure of CiCRY-DASH1 was predicted using AlphaFold2 (Version 2.3.2; https://github.com/google-deepmind/alphafold, accessed on 20 February 2024). Generated models were analyzed for pLDDT, PAE heatmaps, MSA coverage, and LDDT scores. Model 2, with the highest confidence, was selected as the final predicted structure. Structural comparisons with homologous proteins SsCRY (PDB: 1NP7) and AtCRY (PDB: 2VTB) were performed using PyMOL 3.10 software (Schrödinger, New York, NY, USA).

### 3.9. In Vitro and In Vivo Activity Assays of CiCRY-DASH1

#### 3.9.1. Activity Assay of CiCRY-DASH1 in *E. coli*

BL21-pET28a-CiCRY-DASH1 and BL21-pET-28a(+) strains were cultured and induced with IPTG for 8 h as described above. Bacterial cultures were serially diluted and spread onto LB agar plates. To assess UV resistance, plates were exposed to UVB irradiation for 2, 4, and 6 min with lids removed. Following irradiation, plates were incubated overnight at 37 °C. For dark repair assays, plates were immediately wrapped in aluminum foil to prevent photoreactivation; for light repair assays, plates were incubated under white light. Experiments were performed in triplicate for each condition. Bacterial survival was quantified by directly counting the number of CFU on each plate.

#### 3.9.2. Assay of CiCRY-DASH1 Repair Activity Towards CPD Under White and Blue Light

Repair experiments were conducted on single-stranded DNA Oligo(dT)_16_ containing pyrimidine dimers under white and blue light irradiation. The reaction system consists of 50 μL of 0.01 mol/L DTT, 50 μL of 4 × 10^−4^ mol/L Oligo(dT)_16_, and 500 μL of repair buffer (50 mM Tris/Cl, 50 mM NaCl, 1 mM EDTA, 10% (*w*/*v*) glycerol, pH 7.4). The system was placed in quartz cuvettes, and absorbance at 260 nm was continuously monitored every 10 min under UVB irradiation (100 μW/cm^2^) until a stable value was reached, indicating UV-induced DNA damage and CPD substrate formation. The formation of photoproducts was monitored by the characteristic decrease in absorbance at 260 nm, resulting from the loss of the pyrimidine conjugated double-bond system. Irradiation was strictly terminated when the absorbance reached a consistent, predetermined baseline plateau, ensuring comparable starting levels of DNA damage across all samples before enzyme addition. Subsequently, 100 μL of purified CiCRY-DASH1 protein was added to the experimental group, and 100 μL of bovine serum albumin (BSA) was added to the control group. Repair experiments were performed in parallel under white or blue light, with A_260_ measured over time. Each group was performed in triplicate. The distance from the light source to the reaction mixture was fixed at 10 cm to ensure consistent irradiance.

#### 3.9.3. Repair Activity Assay of CiCRY-DASH1 on 6-4PPs

The method reported by Li [39] was modified to assess CiCRY-DASH1 repair activity on 6-4PPs. Briefly, the 5′-GTATTATG-3′ oligonucleotide was prepared in ddH_2_O to a 400 μM working solution, placed in a quartz cuvette, and irradiated with UVB light (100 μW/cm^2^). Absorbance at 260 nm was measured at specified intervals until a stable reading was obtained, indicating the formation of 6-4PPs as the repair substrate. Purified CiCRY-DASH1 was incubated at 15 °C with 10 mM DTT and activated under blue light (450–460 nm) for 30 min. A 1:3 ratio of CiCRY-DASH1 to 6-4PPs was prepared in repair buffer, and the repair reaction was performed under blue light irradiation. A_260_ was recorded at specific intervals using a UV spectrophotometer to monitor the repair process.

### 3.10. Effect of the Loop Region of CiCRY-DASH1 on Photorepair Activity

#### 3.10.1. Construction of Loop-Truncated Plasmids

To investigate the functional roles of the N-terminal and C-terminal loop regions of CiCRY-DASH1, partial or complete deletions were introduced. Coding sequences corresponding to CRY_1–610, CRY_46–610, CRY_1–560, and CRY_46–560 were cloned into the pED expression vector, fused with an N-terminal 6×His-GST tag. An HRV 3C protease recognition site was inserted between the tag and the target protein for tag removal. The resulting recombinant plasmids were named 01_pED-6His-GST-pp-CRY_1-610, 02_pED-6His-GST-pp-CRY_46–610, 03_pED-6His-GST-pp-CRY_1–560, and 04_pED-6His-GST-pp-CRY_46–560. PCR and sequencing verified all constructs.

#### 3.10.2. Protein Expression Assay

The four recombinant plasmids were individually transformed into *E. coli* BL21(DE3) competent cells. Single colonies were inoculated into 5 mL of LB medium supplemented with 50 μg/mL ampicillin and cultured at 37 °C until the OD_600_ reached 0.6–0.8. Protein expression was induced with 0.5 mM IPTG, followed by incubation at 16 °C for 12–16 h. Cells were harvested, lysed by ultrasonication, and the lysates were clarified by centrifugation. The soluble fraction was subjected to small-scale purification using glutathione Sepharose resin (binding at 4 °C for 1 h). Bound proteins were released by digestion with HRV 3C protease at 4 °C for 16 h, and eluted fractions were further purified by Ni-NTA affinity chromatography with 10–400 mM imidazole gradient. Fractions containing highly purified 6His-CRY proteins from the 400 mM imidazole eluates were collected, concentrated using 10 kDa centrifugal filters, and stored at −80 °C. SDS-PAGE assessed protein purity and integrity. For preliminary expression analysis, samples were resolved on 12% separating gels. For high-resolution analysis of purified fractions, 4–20% precast polyacrylamide gradient gels (Lablead, Beijing, China) were utilized to ensure optimal resolution of both high- and low-molecular-weight species.

#### 3.10.3. Repair Experiments of CPD and 6-4PPs by CRY Proteins

Using the same methods described in Section 3.9.2 and Section 3.9.3, the repair activity of the four truncated CRY proteins on CPD and 6-4PPs was assessed.

## 4. Conclusions

In conclusion, this study successfully characterized the cryptochrome CiCRY-DASH1 from the Antarctic microalga *Chlamydomonas* sp. ICE-L. Distinct from many previously reported mesophilic homologs, our findings establish CiCRY-DASH1 as a highly efficient bifunctional enzyme capable of managing both CPD and 6-4PPs lesions. This dual-repair capacity provides the essential molecular basis for genomic stability in Antarctic ice algae within their extreme, high-UV habitat.

Beyond baseline characterization, the systematic deletion of the unique N- and C-terminal loops has uncovered critical general laws governing the catalytic regulation of this enzyme. The N-terminal loop was identified as an intrinsic regulatory constraint; its removal significantly enhances repair efficiency for both types of lesions, a mechanism that likely mirrors the autoinhibition observed in higher-plant cryptochromes. Conversely, the C-terminal loop is indispensable specifically for the monomerization of 6-4PPs. Its absence leads to a complete loss of 6-4PPs repair activity, confirming its critical role in facilitating the complex ‘base flipping’ mechanism required for processing structurally distorted lesions.

Collectively, the exploration of such Antarctic extremozymes significantly expands the pipeline of marine drugs. Our results highlight the potential of harnessing these marine bioactive proteins for the development of advanced dermatological formulations and cancer-preventive interventions. By identifying these ‘inhibitory’ and ‘essential’ structural motifs, this work provides a strategic molecular blueprint for the rational design of high-performance, marine-derived photoprotective therapeutics

## Data Availability

The raw data supporting the conclusions of this article will be made available by the authors on request.

## Supplementary Materials

The following supporting information can be downloaded at: https://www.mdpi.com/article/10.3390/md24010025/s1. Table S1: Composition of Provasoli culture-medium reserve; Table S2: Construction information of Antarctic ice algae CiCRY-DASH1 protein plasmid; Figure S1: Colony PCR electrophoretic map of pET-28a-*CiCRY-DASH1*; Figure S2: Five prediction models of CiCRY-DASH1 protein. (A) Model_1; (B) Model_2; (C): Model_3; (D): Model_4; (E): Model_5; Figure S3: Five prediction models predict alignment errors; Figure S4: The distribution map of the coverage area of multiple sequence alignments; Figure S5: The local distance difference test evaluates the local accuracy of the predicted structure; Figure S6: Schematic diagram of CiCRY-DASH1 plasmid construction; Figure S7: Sequencing verification results of 01_pED-6His-GST-pp-CRY_1–610; Figure S8: Sequencing verification results of 02_pED-6His-GST-pp-CRY_46–610; Figure S9: Sequencing verification results of 03_pED-6His-GST-pp-CRY_1–560; Figure S10: Sequencing verification results of 04_pED-6His-GST-pp-CRY_46–560; Figure S11: Test results of 01_pED-6His-GST-pp-CRY_1–610 expression of Ni filler; Figure S12: Test results of 02_pED-6His-GST-pp-CRY_46-610 expression of Ni filler; Figure S13: Test results of 03_pED-6His-GST-pp-CRY_1-560 expression of Ni filler; Figure S14: Test results of 04_pED-6His-GST-pp-CRY_46-560 expression of Ni filler.

## Abbreviations

The following abbreviations are used in this manuscript:

CRYs	Cryptochromes
ORF	Open reading frame
CPD	Cyclobutane pyrimidine dimer
6-4PPs	6-4 photoproducts
CPF	Cryptochrome/Photolyase family
CRY-DASH	DASH-type cryptochromes
LB	Luria–Bertani
IPTG	Isopropyl-β-D-thiogalactoside
pLDDT	Predicted Local Distance Difference Test
PAE	Predicted Alignment Error
MSA	Multiple Sequence Alignment
LDDT	Local Distance Difference Test
CFU	Colony-Forming Units
