# Supplementary material for: Antarctic Microalga Chlamydomonas sp. ICE-L Cryptochrome CiCRY-DASH1 Mediates Efficient DNA Photorepair of UV-Induced Cyclobutane Pyrimidine Dimer and 6-4 Photoproducts
Source: Mar Drugs. 2026 Jan 7;24(1):25. doi: 10.3390/md24010025 (PMC12843461; doi:10.3390/md24010025)
Supplement: Supplementary file 1 [file marinedrugs-24-00025-s001.zip › marinedrugs-4038131-supplementary.pdf]

supplementary material

Supplementary Table

Table S1. Composition of Provasoli culture-medium reserve

Stock solution	Component	Concentration（g/L）
N	NH_4NO_3	23.5
P	$NaH_2PO_4 \cdot 2H_2O$	3.89
II	$Fe(NH_4)_2(SO_4)_2 \cdot 6H_2O$	0.701
	Na_2EDTA	0.66
III	Na_2EDTA	1.0
	H_3BO_3	1.14
	$FeCl_3 \cdot 6H_2O$	0.049
	$MnCl_2 \cdot 4H_2O$	0.146
	$ZnSO_4 \cdot 7H_2O$	0.0022
	$CoCl_2 \cdot 6H_2O$	0.004

Table S2. Construction information of Antarctic ice algae CiCRY-DASH1 protein plasmid

Protein name	Note	Tags	protease	expression vector	recombinant plasmid	expression system
CRY_ 1-610	Full length	N-6His-GST-ppase	ppase	pED	01_pED-6His-GST-pp-CRY_ 1-610	*E.coli*
CRY_ 46-610	truncated	N-6His-GST-ppase	ppase	pED	02_pED-6His-GST-pp-CRY_ 46-610	*E.coli*
CRY_ 1-560	truncated	N-6His-GST-ppase	ppase	pED	03_pED-6His-GST-pp-CRY_ 1-560	*E.coli*
CRY_ 46-560	truncated	N-6His-GST-ppase	ppase	pED	04_pED-6His-GST-pp-CRY_ 46-560	*E.coli*

Supplementary Figures

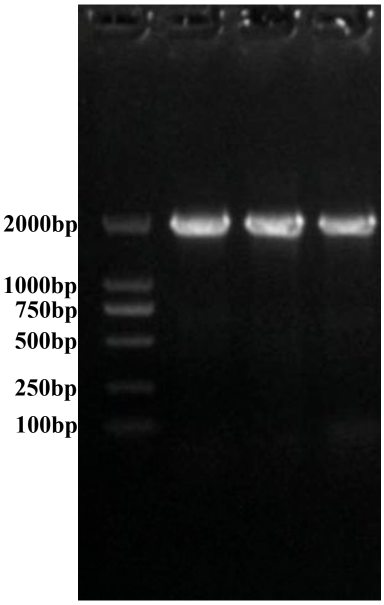

Figure S1. Colony PCR electrophoretic map of pET-28a-*CiCRY-DASH1*.

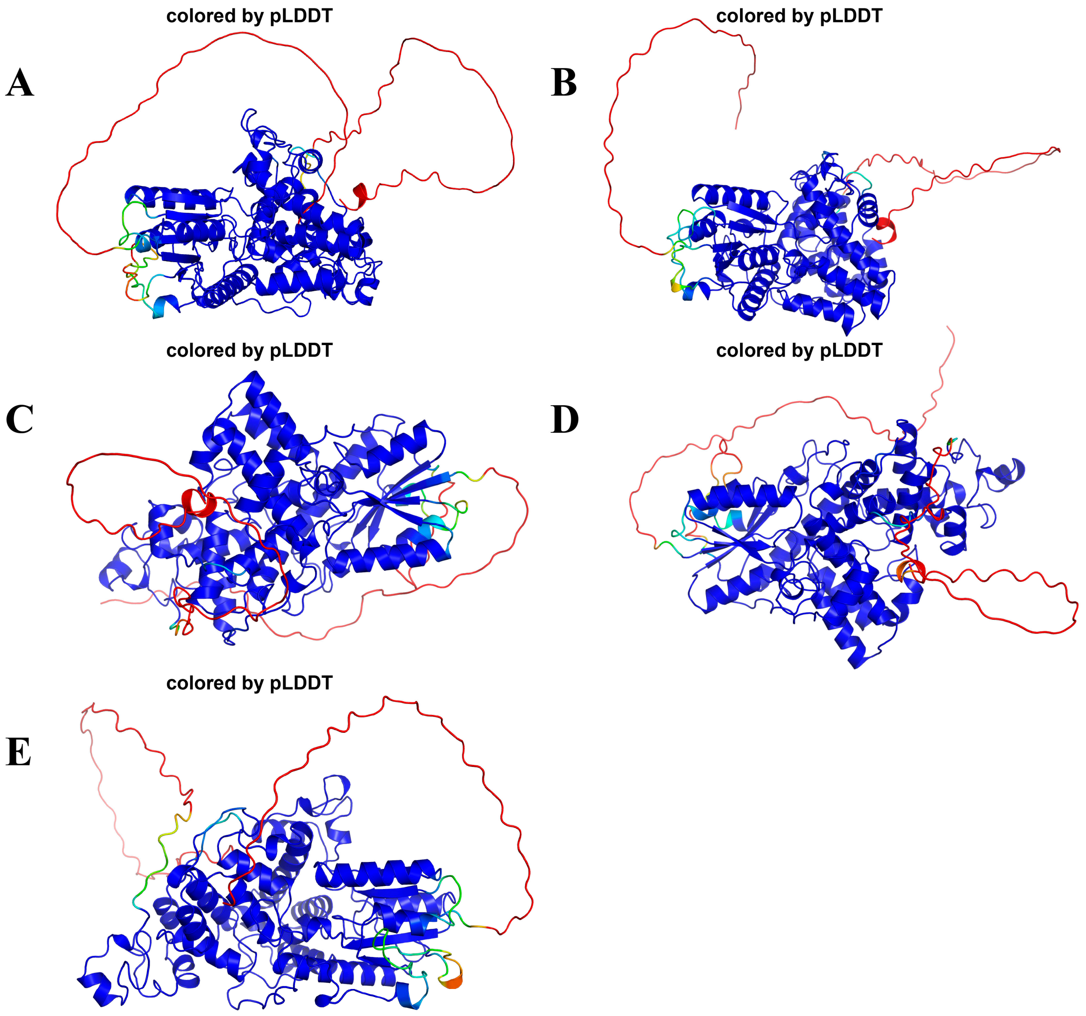

Figure S2. Five prediction models of CiCRY-DASH1 protein. (**A**) Model_1;(**B**)Model_2; (**C**): Model_3; (**D**): Model_4; (**E**): Model_5.

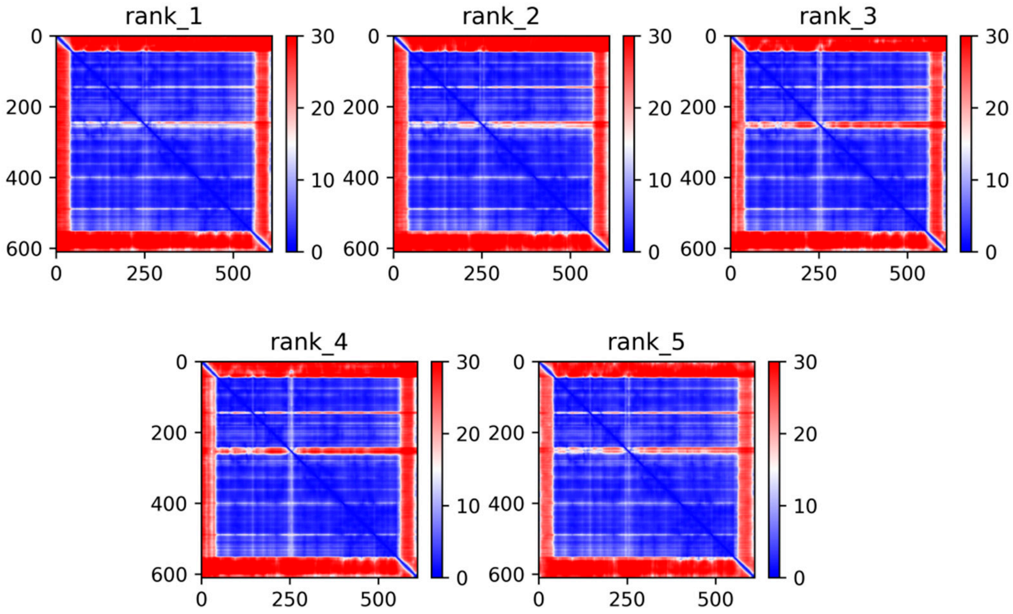

Figure S3. Five prediction models predict alignment errors.

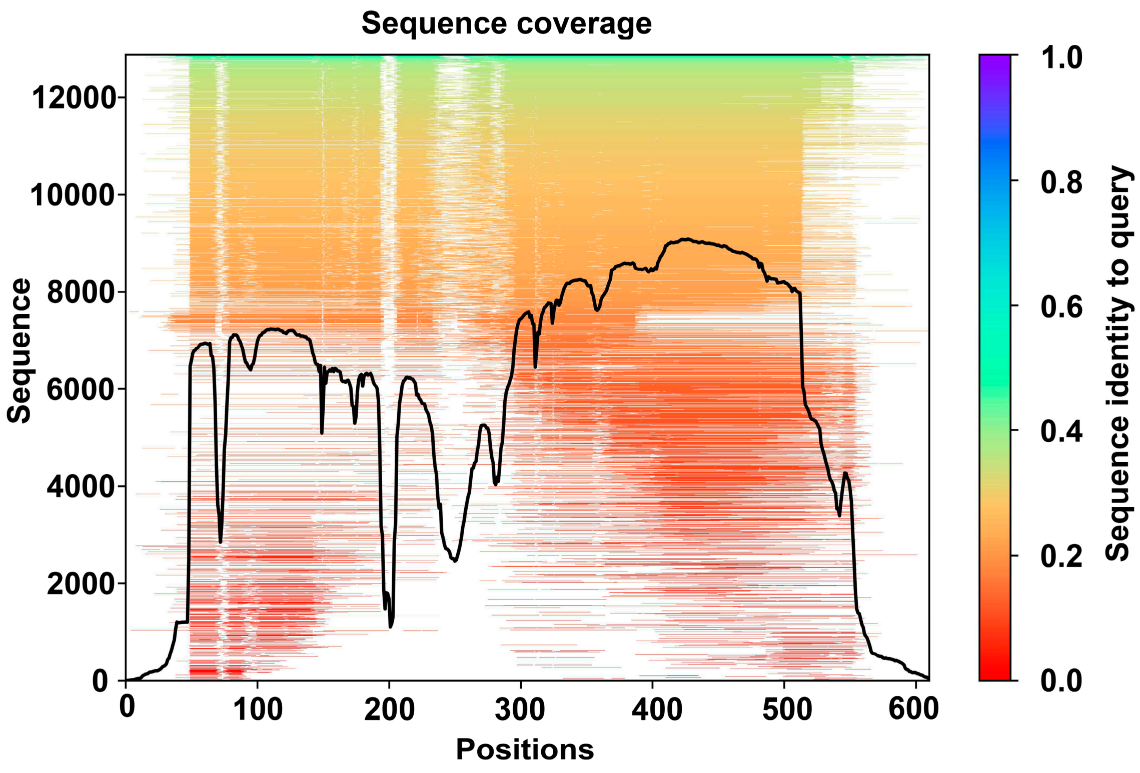

Figure S4. The distribution map of the coverage area of multiple sequence alignments.

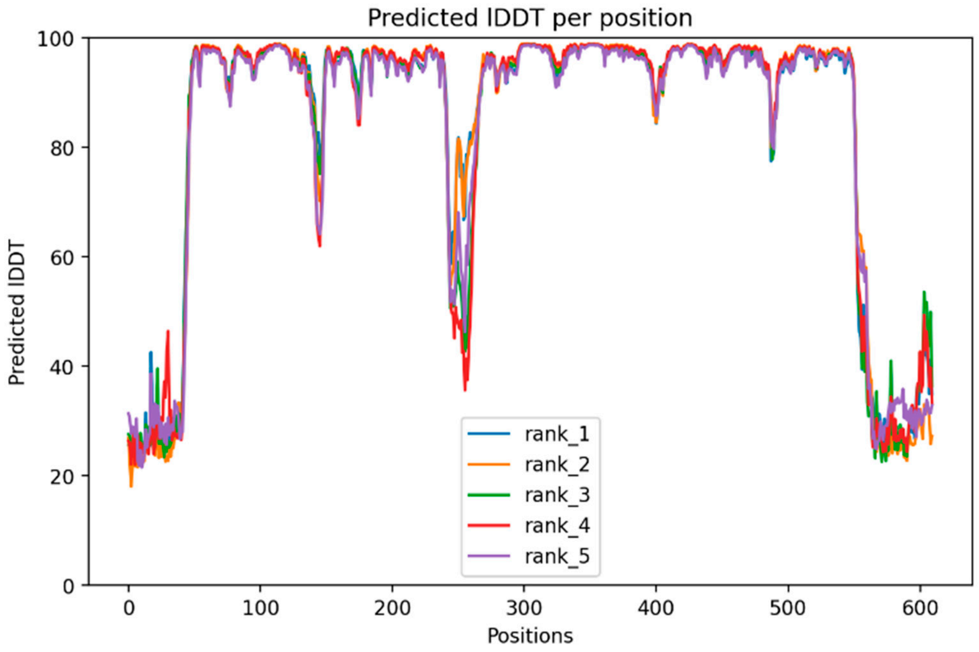

Figure S5. The local distance difference test evaluates the local accuracy of the predicted structure.

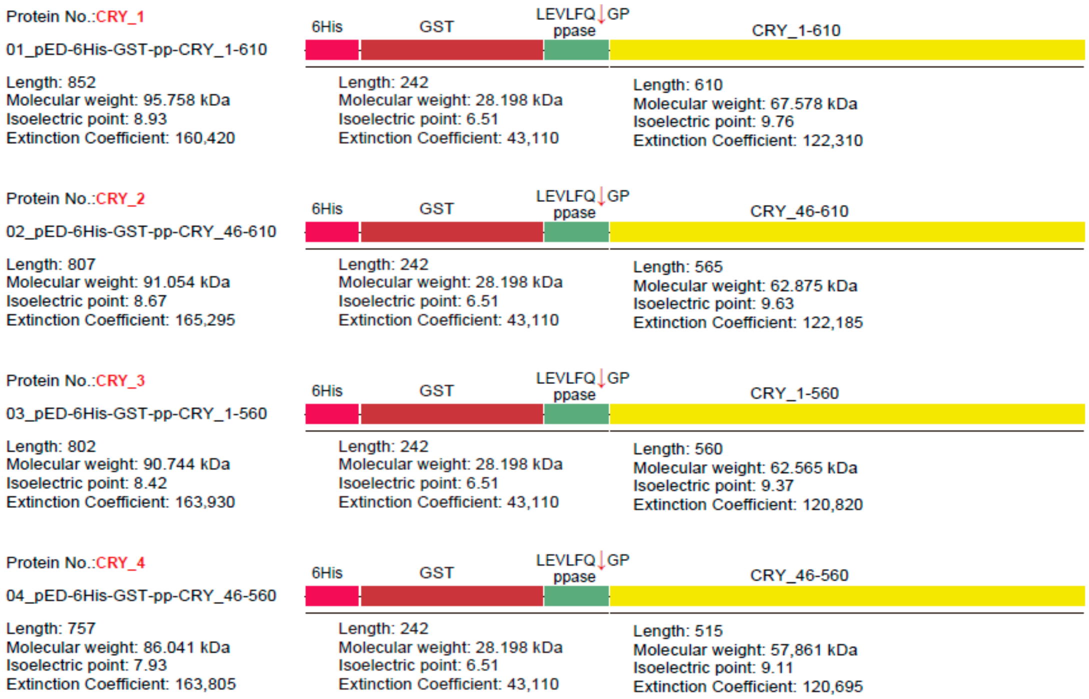

Figure S6. Schematic diagram of CiCRY-DASH1 plasmid construction.

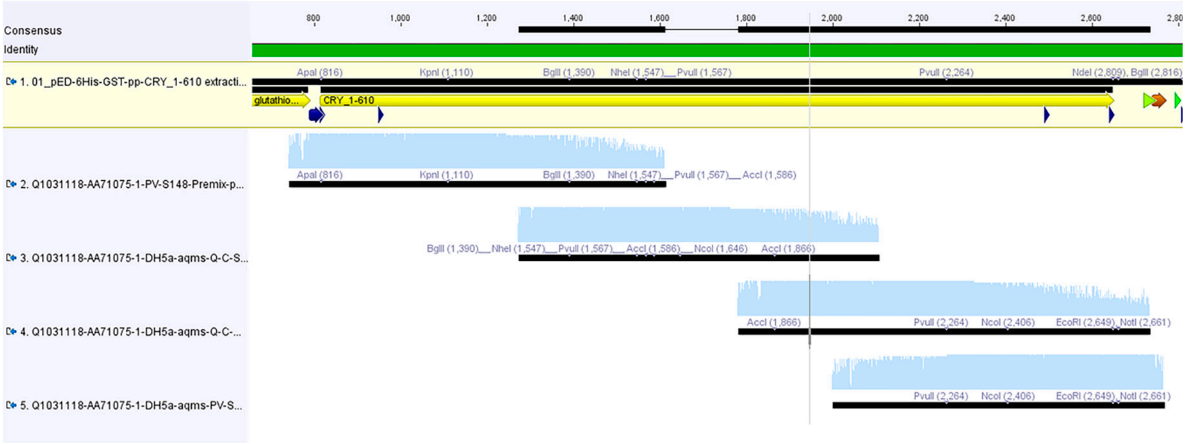

Figure S7. Sequencing verification results of 01_pED-6His-GST-pp-CRY_1–610.

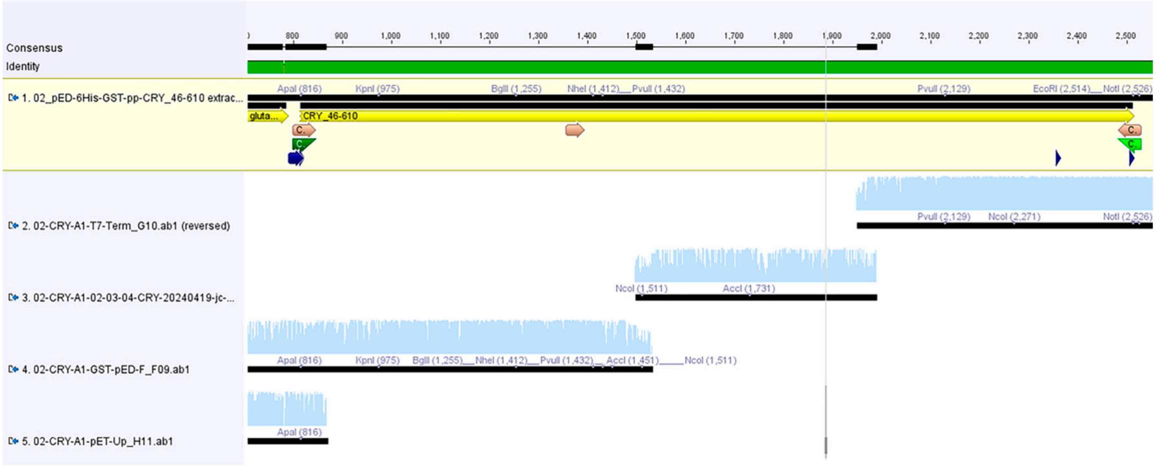

Figure S8. Sequencing verification results of 02_pED-6His-GST-pp-CRY_46–610.

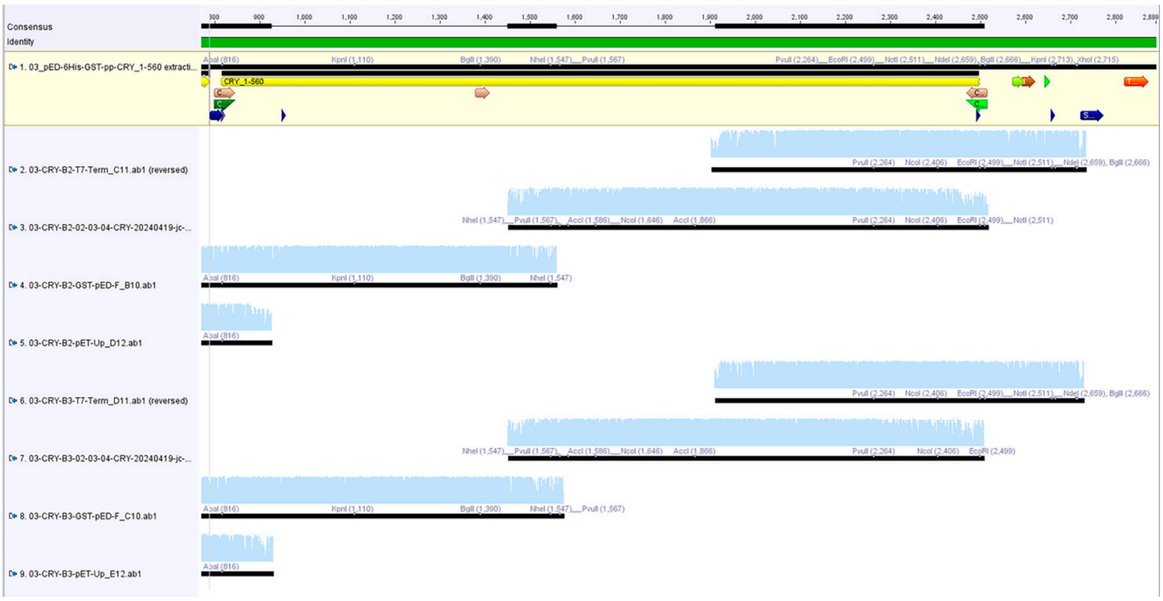

Figure S9. Sequencing verification results of 03_pED-6His-GST-pp-CRY_1–560.

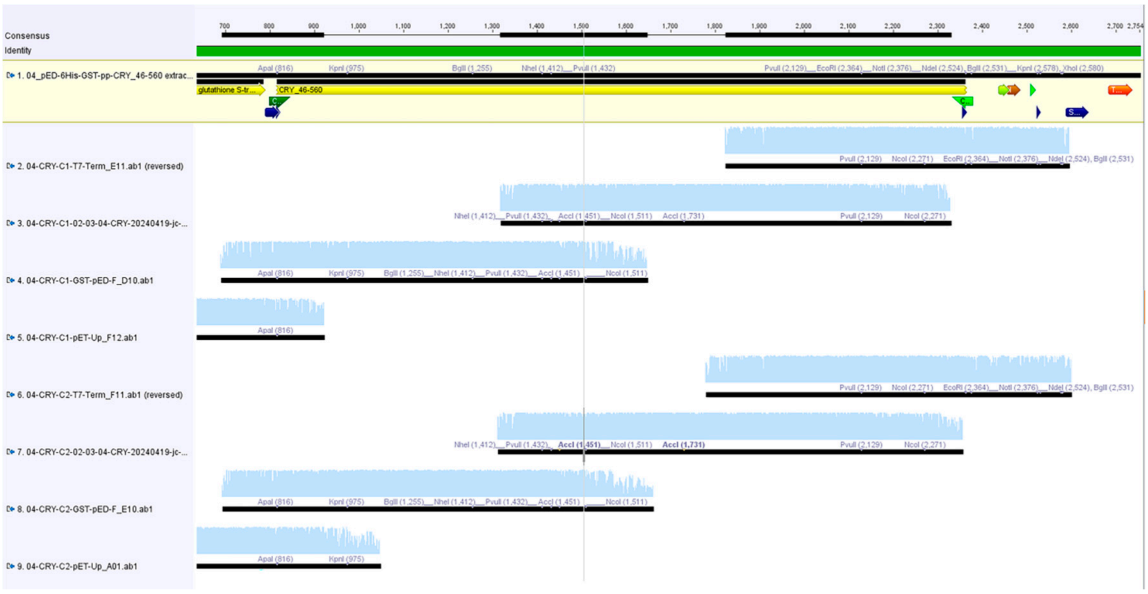

Figure S10. Sequencing verification results of 04_pED-6His-GST-pp-CRY_46–560.

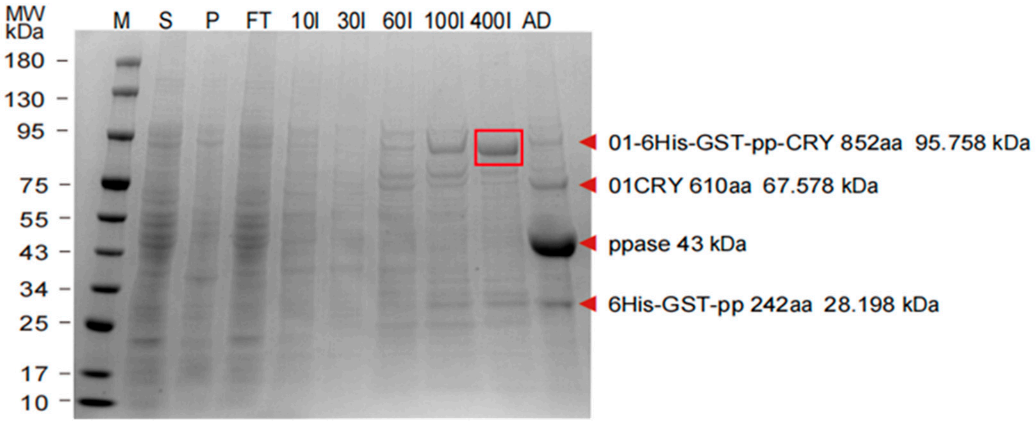

Figure S11. Test results of 01_pED-6His-GST-pp-CRY_1–610 expression of Ni filler.

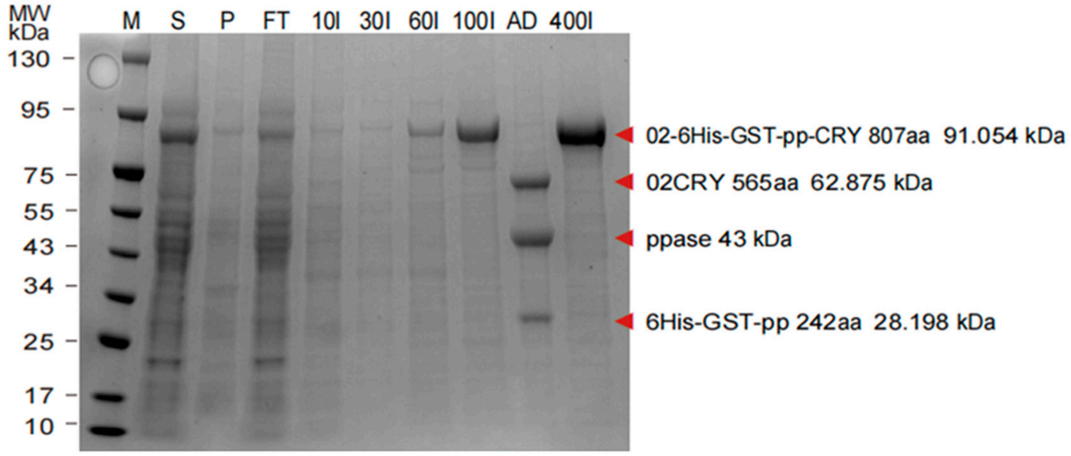

Figure S12. Test results of 02_pED-6His-GST-pp-CRY_46-610 expression of Ni filler.

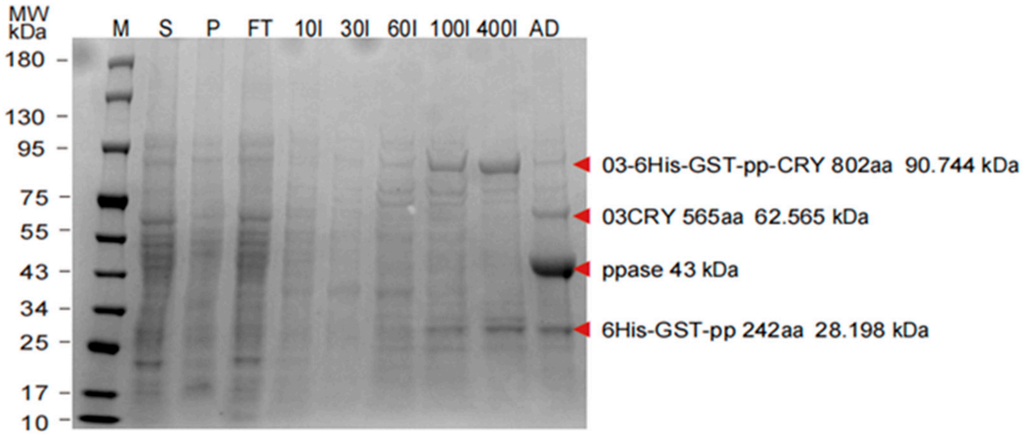

0

Figure S13. Test results of 03_pED-6His-GST-pp-CRY_1-560 expression of Ni filler.

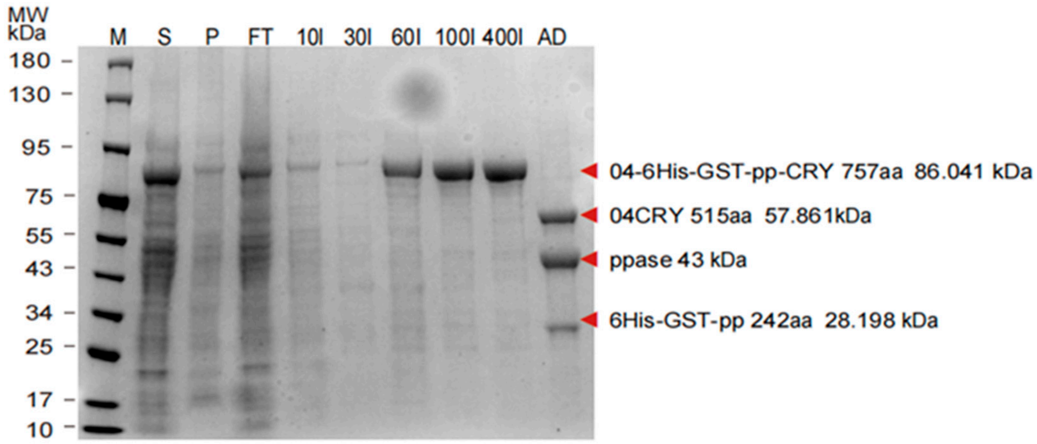

Figure S14. Test results of 04_pED-6His-GST-pp-CRY_46-560 expression of Ni filler.
